# Analysis of clinical characteristics and risk factors for patients with heat stroke in western China in 2022: a multicenter retrospective study

**DOI:** 10.3389/fmed.2025.1467771

**Published:** 2025-01-22

**Authors:** Xin Zheng, Yuchun Gao, Qinli Xie, Qiulan Chen, Chuan Guo, Qionglan Dong, Jin Tang, Jun Luo, Ying Ge, Jian He, Xiaolin Hou, Guanghong Zhou, Yuan Chen, Haiquan Cao, Jiujia Xiao, An Lan, Qiu Chen, Yonghong Zeng, Jing Huang, Huaicong Long

**Affiliations:** ^1^Department of Geriatric Intensive Care Unit, Sichuan Provincial People's Hospital, University of Electronic Science and Technology of China, Chengdu, China; ^2^Department of Physical Examination Center, Chongqing Emergency Medical Center, Chongqing Key Laboratory of Emergency Medicine, Chongqing University Central Hospital, Chongqing, China; ^3^Department of Critical Care Medicine, Nanchong Hospital of Beijing Anzhen Hospital, Capital Medical University, Nanchong Central Hospital, The Second Clinical Medical College of North Sichuan Medical College, Nanchong, China; ^4^Department of Critical Care Medicine, The First Affiliated Hospital of Chengdu Medical College, Chengdu, China; ^5^Department of Critical Care Medicine, The Third Hospital of Mianyang, Mianyang, China; ^6^Department of Critical Care Medicine, Clinical Medical College, Affiliated Hospital of Chengdu University, Chengdu, China; ^7^Department of Critical Care Medicine, Xuanhan County People's Hospital, Dazhou, China; ^8^Department of Critical Care Medicine, Affiliated Hospital of North Sichuan Medical College, Nanchong, China; ^9^Department of Pulmonary and Critical Care Medicine, Chongqing General Hospital, Chongqing, China; ^10^Emergency Medical Department, The First People’s Hospital of Zigong, Zigong, China; ^11^Department of Respiratory and Critical Care Medicine, Sichuan Provincial People's Hospital, University of Electronic Science and Technology of China, Chengdu, China

**Keywords:** heat stroke, prognosis, clinical characteristics, risk factor, treatment

## Abstract

**Objectives:**

To analyzed the clinical characteristics and treatment modalities of heat stroke (HS) and to identify risk factors for a poor prognosis of HS and provide reference suggestions for its treatment and prevention.

**Measurements and main results:**

We enrolled a total of 247 patients, with hypertension, diabetes, and psychosis being the top three comorbidities associated with HS. The incidence of HS was higher among males and older individuals. Compared to the control group, the poor prognosis group experienced higher temperatures, a higher incidence of cerebral edema, and gastrointestinal bleeding (all *p* < 0.05). The poor prognosis group had significantly higher blood pH, HCO3-, Lac, Scr, AST, ALT, DBIL, CKMB, PT, DD, and PLT (all *p* < 0.05). Furthermore, logistic regression analysis revealed that Lac, Scr, and APACHE II were risk factors for poor prognosis (*p* < 0.05). The AUC values for the combined diagnostic model were 0.848 (95% CI: 0.781–0.914). Male morbidity, the number of patients with combined hypertension, the prognosis, and the APACHE II score and ALT level were all greater (*p* < 0.05) in the CHS group. The Kaplan–Meier analysis revealed that the CHS group had a significantly higher mortality rate than the EHS group.

**Conclusion:**

A high incidence of hypertension, diabetes, psychosis, men, and older persons may be associated with HS. HS patients with high blood cell counts, impaired coagulation, liver and kidney diseases, and those with a specific type of CHS may face a poor prognosis. In patients with heart failure, APACHE II, Lac, and Scr were independent risk factors for a poor prognosis.

## Background

Humans are experiencing unprecedented heat exposure due to the increased frequency, duration and intensity of extreme heat events (1). Temperatures are expected to continue to rise, and if global warming continues at this rate, up to 1.2 billion people worldwide could be at risk of HS each year by 2,100 (2).

HS is a severe heat-related injury (3, 4). This type of heat injury is particularly dangerous because it occurs when the body’s core temperature exceeds 40°C, leading to organ dysfunction, hot skin, and disorientation (5, 6). There are two types of HS: exertional heat stroke (EHS) and classic heat stroke (CHS). CHS primarily affects elderly individuals, children, frail people, and those with chronic illnesses (7). EHS is often seen in young, healthy individuals after intense physical activity or training (8, 9). The mortality rate for exhaustion in critical care was 26.5%, while the mortality rate for CHS was 63.2%.

Early identification and treatment can reduce the morbidity and mortality rates of HS, which can potentially be prevented (10, 11). However, there is currently limited research focusing on HS in large samples and multiple centers, and our understanding of HS is not yet systematic or thorough. Additionally, due to the influence of the Tibetan Plateau, western China, Sichuan Province and Chongqing city experienced a very hot summer in 2022, which led to a high incidence of HS. This paper presents the results of a retrospective study in which we examined the clinical features and treatment approaches of HS in order to determine the risk factors associated with a poor prognosis and to offer treatment and preventative recommendations.

## Methods

### Research population

The study population originated from a multicenter study conducted by the Sichuan Academy of Medical Sciences & Sichuan Provincial People’s Hospital, focusing on the clinical characteristics and risk factors of patients with HS. In 2022, patients with a definitive diagnosis of HS were recruited from 10 large tertiary care hospitals. This was a retrospective study, thus no informed consent forms were required from participants, and clinical registration was also not necessary. The study was approved by the Medical Ethics Committee of Sichuan Academy of Medical Sciences·Sichuan Provincial People’s Hospital (protocol number: 2023–57, approval date: February 17, 2023), and conducted in accordance with Good Clinical Practice and the principles of the Declaration of Helsinki.

**Figure 1:**
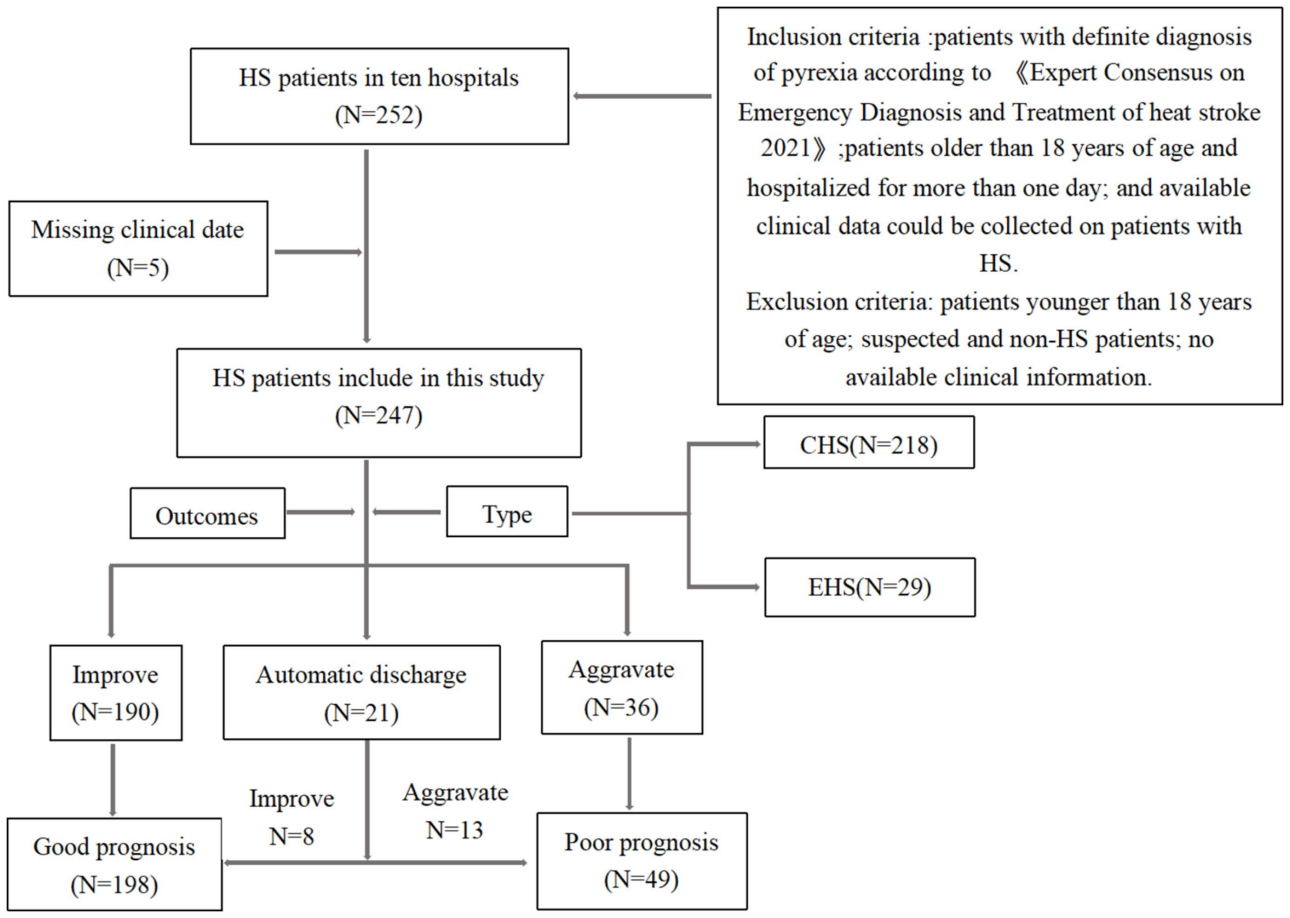
Flowchart for patient enrollment.

The inclusion criteria were as follows (12, 13): (1) patients who were exposed to high environmental temperature. (2) presenting with one or more of the following: disseminated intravascular coagulation, CNS manifestation (impaired consciousness, cerebellar symptoms, convulsive seizures), or hepatic or renal dysfunction. (3) patients older than 18 years of age, hospitalized for more than one day, and with HS and available clinical data for collection. The exclusion criteria were as follows: The exclusion criteria were as follows: patients younger than 18 years of age, suspected non-HS patients, and patients lacking available clinical information. Detailed information on the included participants is shown in Figure 1.

### Data extractions

We extracted study variables including demographic characteristics such as age, gender, location of onset, length of hospitalization, length of ICU stay, and respiratory rate (R), APACHE II score, Place onset, comorbidities, and symptoms. Initial laboratory data collected included arterial blood gas analysis, white blood cells (WBC), platelets (PLT), hemoglobin (HB), serum creatinine (Scr), blood urea nitrogen (BUN), aspartate transferase (AST), alanine aminotransferase (ALT), bilirubin direct (DBIL), indirect bilirubin (TBIL), myoglobin (MYO), CKMB, troponin (cTn), B-type brain natriuretic peptide (BNP), D-dimer (D-D), fibrin (FIB), prothrombin time (PT), and activated partial thromboplastin time (APTT). Additionally, therapeutic interventions were also recorded.

### Patient grouping

Patients were grouped into two groups according to their outcomes, death or poor prognosis patients were divided into poor prognosis group. The prognosis of discharged patients was followed up via telephone interviews. Based on the classification of HS patients, they were divided into the CHS group and the EHS group.

### Statistical analysis

SPSS 25.0 statistical software was used to process the data. Count data were analyzed using the chi-square test (χ^2^). Measurement data were analyzed using the t-test and the Mann–Whitney U test. Missing values were addressed with regression modeling. Multivariate logistic regression analysis was conducted to identify potential risk factors in the poor prognosis group of HS patients. ROC curves were plotted, and the predictive accuracy of each index for poor prognosis in HS patients was evaluated by calculating the AUC and 95% CI. A *p*-value of <0.05 was considered statistically significant. Kaplan–Meier survival analysis was performed to compare the prognosis between patients in the CHS group and the EHS group.

## Results

### General characteristics

A total of 247 patients with a confirmed diagnosis of HS were ultimately included in this study. The geographic distribution of the patients in Sichuan Province and Chongqing is depicted in Supplementary Figure S1. The mean age of the patients (standard deviation) was 68.07 ± 14 years, and the percentage of males was 61.5% (152/247). The majority of the patients fell within the 51–90 age range, with the peak number being in the 61–80 age group (Supplementary Figure S2). The percentages of patients with comorbidities such as hypertension, diabetes, psychosis, stroke, COPD, Parkinson’s disease and rheumatic disease were 31.6% (78/247), 15.4% (38/247), 13.8% (15/247), 13.4% (33/247), 7.7% (19/247), 3.2% (8/247) and 3.2% (8/247), respectively. The in-hospital mortality rate among HS patients was 14.57%.

### Characteristics of the groups

There were 198 (119 males, 79 females) in the good prognosis group, and the average age was 67.27 ± 15.45 years. There were 49 (33 males, 16 females) in the poor prognosis group, and the average age was 71.29 ± 10.55 years. The poor prognosis group exhibited more gastrointestinal symptoms, encephaledema, comorbid psychiatric disease, higher temperature at admission, longer hospitalization, and higher APACHE II scores. Based on the definition, heatstroke is classified into two types: Men in the EHS group exhibited a higher incidence and were at a greater risk of developing the disease outdoors. The CHS group had a higher proportion of patients with hypertension and higher APACHE II scores (*p* < 0.05) (Table 1).

**Table 1:** Comparison of the general information of the different groups of patients.

	Good prognosis (*n* = 198)	Poor prognosis (*n* = 49)	*p* value	CHS (*n* = 218)	EHS (*n* = 29)	*P* value
Sex						
Male	119 (60.1%)	33 (61.5%)	0.351	128 (58.7%)	24 (82.8%)	0.012*
Female	79 (39.9%)	16 (38.5%)		90 (41.3%)	5 (17.2%)	
Age (years)	67.27 (15.45)	71.29 (10.55)	0.087	79.89 (12.19)	46.90 (14.60)	0.064
Place onset						
Indoor	124 (64.2%)	37 (75.5%)	0.136	160 (74.4%)	1 (3.7%)	<0.001*
Outdoor	69 (35.8%)	12 (24.5%)		55 (25.6%)	26 (96.3%)	
Temperature (°C)	39.6 (38.6,40.8)	40.1 (39.1,41.05)	0.027*	39.9 (38.8,40.8)	39.0 (37.5,40.50)	0.05
T in 24 h (°C)	36.4 (36.1,36.7)	36.4 (36.0,37.3)	0.145	36.4 (36.2,36.7)	36.3 (36.0,36.6)	0.225
T in 48 h (°C)	36.5 (36.2,36.7)	36.5 (36.0,37.1)	0.998	36.5 (36.2,36.7)	36.4 (36.2,36.6)	0.831
R (time/min)	22 (19,28)	24 (20,30)	0.188	23 (20,28.25)	20 (16.5,30.0)	0.223
Gastrointestinal symptom						
Non	150 (75.8%)	36 (73.5%)	<0.001*	166 (76.1%)	20 (69.0%)	0.142
Nausea and vomiting	18 (9.1%)	0 (0.00%)		13 (6.0%)	5 (17.2%)	
Gastrointestinal hemorrhage	1 (0.5%)	6 (12.2%)		7 (3.2%)	0 (0.0%)	
Distention and diarrhea	29 (14.6%)	7 (14.3%)		26 (11.9%)	4 (13.8%)	
Neurological symptoms						
Non	32 (16.2%)	1 (2.0%)	0.013*	27 (12.4%)	6 (20.7%)	0.47
Disturbance of consciousness	154 (77.8%)	47 (95.9%)		179 (82.1%)	22 (75.9%)	
Dizziness and headache	12 (6.1%)	1 (2.0%)		12 (5.5%)	1 (3.4%)	
Encephaledema	3 (1.6%)	8 (14.3%)	<0.001*	11 (5.2%)	0 (0.0%)	0,238
Cerebrovascular accident	22 (11.7%)	10 (17.5%)	0.252	31 (14.4%)	1 (3.4%)	0.079
Comorbidity						
Diabetes	31 (15.7%)	7 (14.3%)	0.802	36 (16.6%)	2 (6.9%)	0.272
Hypertension	65 (32.8%)	13 (26.5%)	0.396	75 (34.4%)	3 (10.3%)	0.010*
Parkinson’s	7 (3.5%)	1 (2.0%)	0.506	8 (3.7%)	0 (0.0%)	0.601
Psychosis	7 (3.5%)	8 (16.3%)	0.001*	14 (6.4%)	1 (3.4%)	1
COPD	12 (6.1%)	7 (14.3%)	0.053	19 (8.7%)	0 (0.0%)	0.14
Stroke	24 (12.1%)	9 (18.4%)	0.25	32 (14.7%)	1 (3.4%)	0.143
Infection						
Non	121 (61.4%)	33 (68.8%)	0.0694	141 (65.3%)	13 (44.8%)	0.138
Pulmonary infection	69 (35.0%)	13 (27.1%)		67 (31.0%)	15 (51.7%)	
Urinary infection	2 (1.0%)	1 (2.1%)		3 (1.4%)	0 (0.0%)	
Bloodstream infection	5 (2.5%)	1 (2.1%)		5 (2.3%)	1 (3.4%)	
Length of hospitalization stay (days)	7 (3，12)	3 (1，9.5)	0.011*	7 (2，11)	6 (3，14)	0.283
Length of ICU stay (days)	3 (1，6)	2 (1，9)	0.2	3 (1，6)	3 (1，6)	0.99
Onset-to-salvage time (h)	4 (2，7.25)	5 (2.5，9)	0.202	4 (2，8)	3 (2，7.5)	0.202
APACHE II score	21 (16,27)	30 (25,35)	<0.001*	24 (18,29)	18 (14,27)	0.012*

T in 24 h indicates the lowest T within the first 24 h, T in 48 h indicates the lowest T within the first 48 h; *Indicates a *p* value<0.05 compared with the good prognosis group or EHS group.

In the laboratory tests of the two groups, the poor prognosis group exhibited higher values of HCO3-, Lac, Scr, BUN, AST, ALT, DBIL, CKMB, PT, APTT, and D-D, while the values of pH and PLT were lower than those in the good prognosis group (*p* < 0.05). The differences in the remaining data between the two groups were not statistically significant (*p* > 0.05). Additionally, we observed that ALT levels in the CHS group were statistically higher than those in the EHS group (*p* < 0.05) (Table 2).

**Table 2:** Comparison of the clinical laboratory test results between the different groups of patient.

	Good prognosis (*n* = 198)	Poor prognosis (*n* = 49)	*P* value	CHS (*n* = 218)	EHS (*n* = 29)	*P* value
pH	7.44 (0.10)	7.38 (0.12)	<0.001*	7.43 (0.10)	7.41 (0.10)	0.765
PaCO_2_ (mmHg)	27.11 (22.72,33.10)	27.00 (21.20,36.75)	0.805	27.06 (22.38,33.61)	26.70 (22.90,32.10)	0.954
PaO_2_ (mmHg)	104.50 (77.00,133.00)	89.00 (68.50,120.50)	0.05	103.70 (77.00,132.87)	87.96 (73.53,122.63)	0.39
HCO3^−^ (mmol/L)	19.15 (16.10,23.00)	15.60 (12.75,19.40)	<0.001*	19.65 (15.00,22.13)	18.40 (16.10,22.50)	0.934
Lac (mmol/L)	3.00 (1.58,4.74)	4.20 (2.70,6.16)	0.002*	3.3 (1.8,5.3)	3.2 (2.1,4.28)	0.431
Scr (umol/L)	97.20 (73.63,160.05)	167.00 (120.00,196.70)	<0.001*	114.54 (78.85,173.00)	106.00 (79.75,172.40)	0.764
BUN (mmol/L)	8.04 (5.79,12.81)	9.78 (7.19,14.36)	0.020*	8.65 (6.00,13.24)	7,23 (6.29,10.34)	0.281
AST (U/L)	45.00 (26.00,120.00)	84.00 (49.50,220.00)	0.001*	51.00 (29.00,132.00)	55.00 (29.00,132.00)	0.335
ALT (U/L)	31.05 (15.75,76.10)	68.00 (28.00,139.50)	0.001*	35.00 (16.00,78.00)	56.00 (27.30,133.50)	0.019*
TBIL (umol/L)	17.75 (12.76,26.30)	21.80 (15.10,31.10)	0.121	18.40 (13.15,27.13)	17.92 (11.80,25.08)	0.453
DBIL (umol/L)	6.75 (4.60,11.38)	10.00 (6.05,14.45)	0.009*	7.00 (4.87,11.95)	7.10 (4.40,13.35)	0.931
MYO (ng/mL)	564.5 (150.95,2103.65)	1200.00 (145.60,6228.34)	0.42	564.5 (150.95,2103.65)	1200.00 (145.60,6228.34)	0.42
cTn (ng/L)	1.41 (0.10,163.67)	6.86 (0.28,597.34)	0.164	1.74 (0.13,171.80)	2.72 (0.07,700.36)	0.939
CKMB (U/L)	10.29 (4.12,25.23)	20.02 (3.73,62.00)	0.018*	10.99 (3.90,28.69)	11.30 (4.71,38.27)	0.706
BNP (pg/mL)	332.35 (84.50,1075.00)	513.90 (131.55,2493.10)	0.64	374.70 (98.85,1230.37)	597.00 (19.15,1917.54)	0.706
PT (s)	14.10 (12.73,15.51)	15.80 (14.03,18.65)	<0.001*	14.45 (12.98,15.90)	14.70 (12.25,16.45)	0.812
APTT (s)	29.25 (25.68,33.63)	31.30 (28.05,37.37)	0.003*	29.25 (26.68,34.50)	30.00 (25.70,33.65)	0.828
FIB (g/L)	2.65 (2.11,3.34)	2.69 (1.90,3.87)	0.824	2.67 (2.11,3.44)	2.49 (1.90,3.10)	0.258
D-D (mg/L)	2.28 (0.85,6.16)	12.76 (5.74,80.08)	<0.001*	3.38 (1.02,12.76)	2.01 (0.78,3.89)	0.08
WBC (1,012/L)	12.03 (9.90,16.03)	12.87 (8.39,18.99)	0.495	12.03 (8.74,16.69)	12.35 (9.06,18.13)	0.461
HB (g/L)	127.50 (21.36)	124.69 (22.38)	0.416	125.69 (21.44)	136.38 (20.31)	0.934
PLT (109/L)	145.50 (96.75,201.25)	103.00 (39.00,161.00)	<0.001*	136.50 (95.00,190.50)	106.00 (61.50,212.50)	0.665
CRP (mg/L)	4.88 (0.58,22.09)	1.95 (0.22,35.26)	0.332	4.87 (0.55,23.44)	3.34 (0.46,33.13)	0.747
PCT (ng/ml)	4.58 (0.78,16.01)	12.15 (1.04,30.07)	0.062	5.55 (0.89,21.71)	5.67 (1.84,15.50)	0.927

^*^Indicates a *p* value < 0.05 compared with the good prognosis group or EHS group. D-D indicate D-dimer.

### Treatment of groups

A greater number of patients in the poor prognosis group underwent fluid replacement, blood purification, sedation, coagulation factor administration, mannitol use, glucocorticoid therapy, and endotracheal intubation compared to those in the good prognosis group (*p* < 0.05). Consequently, mannitol was administered more frequently in the EHS group than in the CHS group (*p* < 0.05). The remaining comparisons between the two groups were not statistically significant (*p* > 0.05) (Supplementary Table S1).

### Risk factors in the poor prognosis group

**Figure 2:**
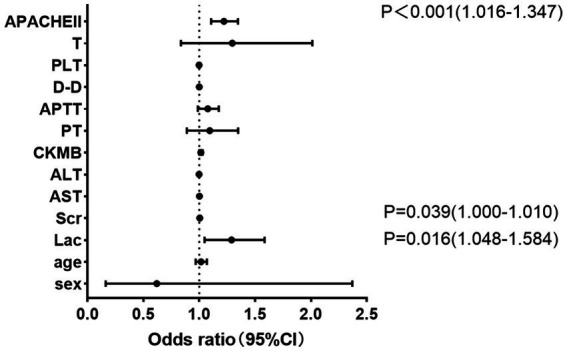
Forest plots for the multifactor logistic regression in the poor prognosis group.

**Figure 3:**
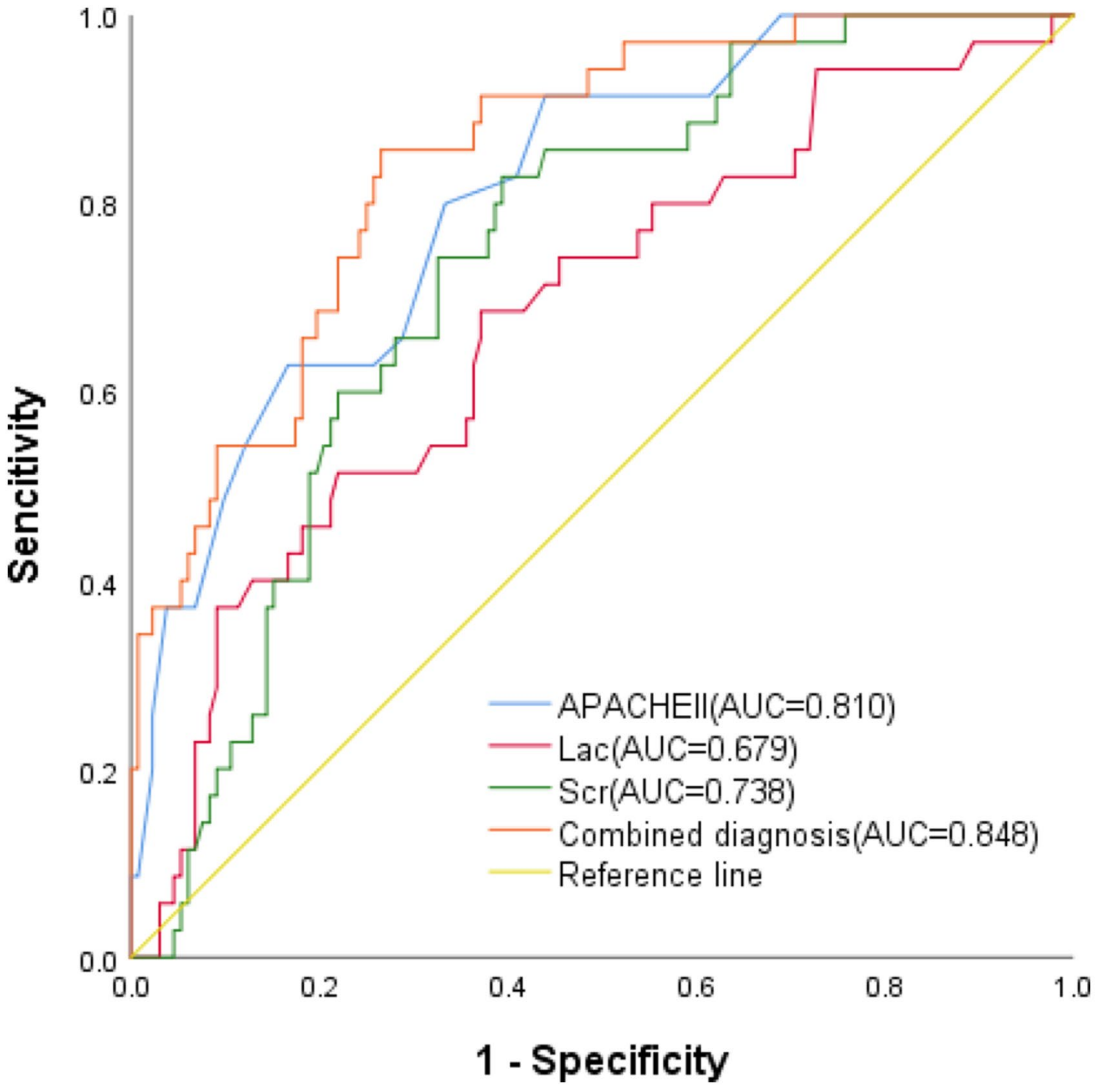
ROC curves for APACHE II, Lac, Scr, and combined diagnosis in the poor prognosis group.

The multivariate logistic regression analyses show that the higher the values of APACHE II, Lac and Scr, the greater the likelihood of a poor prognosis (Supplementary Table S2; Figure 2). The ROC curve demonstrates that the results of APACHE II, Lac and Scr results have better predictive value for patients with poor prognosis (Figure 3; Table 3).

**Table 3:** Comparison of the treatment modalities between the different groups of patients.

	Good prognosis (*n* = 198)	Poor prognosis (*n* = 49)	*P* value	CHS (*n* = 218)	EHS (*n* = 29)	*P* value
Antibiotic	151 (76.3%)	42 (85.7%)	0.152	169 (77.5%)	24 (82.8%)	0.522
Hypothermia	165 (83.3%)	36 (73.5%)	0.112	177 (81.2%)	24 (82.8%)	0.839
Fluid replacement (ml)^#^	1976 (775,2,817)	2,955 (1,500,4,550)	<0.001*	2000 (1,000,3,184)	2,354 (500,3,065)	0.994
Blood purification	10 (5.1%)	9 (18.4%)	0.002*	17 (7.8%)	2 (6.9%)	1
Sedation	86 (43.4%)	39 (79.6%)	<0.001*	109 (50.0%)	16 (55.2%)	0.601
Endotracheal intubation	61 (30.8%)	39 (79.6%)	<0.001*	90 (41.3%)	10 (34.5%)	0.483
Anticoagulant	63 (32.0%)	18 (36.7%)	0.526	74 (33.9%)	7 (25.0%)	0.343
Coagulation factor	17 (8.6%)	22 (44.9%)	<0.001*	33 (15.1%)	6 (20.7%)	0.441
Glucocorticosteroid	49 (24.7%)	19 (38.8%)	0.049*	58 (26.6%)	10 (34.5%)	0.372
Enteral nutrition	76 (38.4%)	26 (46.9%)	0.274	88 (40.4%)	11 (37.9%)	0.801
Mannitol	35 (18.4%)	19 (42.2%)	0.001*	43 (20.8%)	11 (39.3%)	0.029*

^#^Fluid replacement indicates the amount of fluid resuscitation on the first day. *Indicates a *p* value < 0.05 compared with the good prognosis group or EHS group.

### Survival analysis of CHS

We followed 247 patients for survival from admission to day 28. As shown in (Supplementary Figure S3; Supplementary Table S3), the mortality rate in the CHS group was 16.97% (37/218), and the mortality rate in the CHS group was significantly higher than that in the EHS group [0% (0/29)] (*p* < 0.05). Kaplan–Meier analysis revealed that the overall survival rate was higher in the EHS group compared to the CHS group.

## Discussion

Various factors, including environmental conditions such as high temperatures and humidity, comorbidities, aging, delayed heat acclimation, and physical activity, can contribute to the development of hypoxia syndrome (14). Recently, the incidence of HS has increased due to global climate change. Studies indicate that deaths from heatwaves exceeding 35°C increase by 0.45 per 100,000 individuals globally and by 4.7 per 100,000 among individuals aged 64 and older (15, 16). Severe HS is a clinical condition characterized by impaired stress response, which can lead to severe endothelial damage, systemic inflammatory response syndrome, and potentially fatal multi-organ failure (17). Early detection and intervention measures, such as prompt cooling and fluid resuscitation, can decrease the mortality rate associated with HS. Unfortunately, the early signs and symptoms of HS are nonspecific, which makes it difficult to distinguish from other conditions and prolongs the treatment duration (18). However, there is a lack of thorough and organized multicenter research on HS. To reduce the incidence and mortality of HS, we have compiled the clinical features and prognostic factors of the patients included in this study. This will facilitate early detection, timely treatment implementation, and prevention strategies for heatstroke in high-risk populations.

This study included patients with HS from ten tertiary hospitals in various western Chinese locations, such as Chongqing City and Sichuan Province. The clinical data of patients with HS in the groups with good and poor prognoses were compared, revealing that there were more cases in the male group than in the female group, and suggesting that sex hormones and surface area may impact the effectiveness of thermoregulation (19). Sex determines the switch within the temperature-dependent sex determination (TSD) system (20). In the TSD system, Kdm6b-mediated epigenetic modifications function primarily in male sex in the TSD system, while Foxl2 plays a role in female sex determination (20, 21). Furthermore, we discovered that the incidence of heat stroke increases with age, peaking in the 60–80 age group. Earlier research shows that older persons account for the majority of heat-related hospital admissions and fatalities (22, 23). Age-related reductions in thermal effector output during heat stress may cause decreased skin vasodilation in the elderly during heat stress, which may also be associated with alterations in skin microvascular structure, reduced efferent nerve outflow, and defective skin vasodilation signaling (24, 25). Additionally, indoor morbidity was found to exceed outdoor morbidity. Based on our hypothesis, this could be attributed to hot weather, high indoor temperatures, inadequate ventilation, and the higher incidence of indoor morbidity among the elderly, who are more likely to take long-term oral medications that may alter body temperature regulation, such as those for Parkinson’s disease and mental health issues. Fascinatingly, our research also revealed a higher incidence of HS in individuals with underlying medical disorders such diabetes or hypertension. Diabetes involves processes that may affect the vasa nervorum and skin circulation. As neuropathy worsens, it may also increase the risk of experiencing a high fever (26). Individuals with hypertension are more susceptible to heat stress due to the cardiovascular strain and impaired vasoconstriction (27). Although the majority of patients with outdoor morbidity are middle-aged and younger, they often work in hot conditions, such as couriers, porters, and athletes engaged in outdoor training. The poor prognosis group’s body temperature was greater upon admission than the good prognosis group’s, indicating that a high body temperature upon admission can impact a patient’s prognosis. Rehydration therapy and early physical hypothermia reduced the likelihood of a poor patient outcome (*p* < 0.001) (28).

Patients with HS who also suffer from comorbid mental illness may face a challenging prognosis concerning their overall health. HS can affect the thermoregulatory center of the body. Long-term oral antipsychotic medications are necessary for psychiatric patients, and these medications can impair the thermoregulatory centers, increasing the vulnerability of patients to HS (29). According to a meta-analysis, there is a strong correlation between ambient temperature and 10 and 9% increases in the risk of stroke morbidity and fatality, respectively (30). Direct heat impacts particularly are susceptible to causing damage to the central nervous system (CNS), and HS can cause significant CNS damage, manifesting as motor and cognitive impairments, lifelong brain damage, and coma (31–33). Our research shows that patients with concurrent cerebral edema had a poorer outcome. Early mannitol treatment may improve the prognosis of HS patients with concurrent cerebral edema.

In addition to inducing a cytokine storm in response to vascular injury and the coagulopathy underlying the tissue pathology, hyperthermia also elicits an immunological response to platelet-rich clots, initiating a positive feedback loop that results in early multi-organ damage (34). Abnormalities in PLT and coagulation may indicate the severity of HS, and anticoagulant therapy may enhance patient survival. Thermal stimulation has been proposed to cause PLT aggregation and a reduction, or even depletion, of coagulation factors (17). The multifactorial analysis in this study revealed that APACHE II, Lac, and Scr were independent predictors of a poor outcome. Supplementary Table S2 and Figure 2 present the ROC curve analysis for the poor prognosis group. Furthermore, the combined prognostic predictive power of the three indicators was higher, with an AUC value of 0.848 (95% CI: 0.781–0.914) and corresponding sensitivity and specificity values of 0.735 and 0.875, respectively. Another study found AUC values of approximately 0.8 for heart rate, time to normothermia, and SOFA score in predicting severe HS mortality. The results demonstrated that a combination of these three metrics was most effective in predicting 90-day mortality in patients with HS (35). Combining these indicators allows for more accurate evaluation of HS patients’ disease prognosis and enables early modifications to their treatment plan, thereby reducing the likelihood of unfavorable outcomes.

Our study also found that the prognosis of the CHS group was worse than EHS group, and the 28-day mortality rate was also higher than the EHS group. Most patients with CHS are elderly and suffer from numerous comorbidities that are clinically atypical and difficult to identify early. HS often leads to multiple organ dysfunction, and these patients have a low organ reserve capacity. Moreover, the APACHE II scores, Lac levels, and Scr levels of the CHS group were higher than those of the EHS group, indicating a higher risk of mortality. Our study found that aminotransferase levels were slightly elevated in both groups, and the ALT value in the CHS group was higher than that in the EHS group. Liver injury is a common complication of HS. Early HS-related liver injury is mainly manifested by a simultaneous increase in aminotransferase and TBil levels, most of which represent mild liver injury (36). The mechanism of HS-induced liver injury is closely related to systemic inflammatory response, coagulation dysfunction, abnormal liver cell death and abnormal KCs function (37). It has been found that HMOX-1-mediated iron death of KCs (especially KC2) induces liver injury under HS conditions. The regulation of HMOX-1 is a potential therapeutic strategy for HS-related liver injury (38).

The fact that all HS patients were from ten different hospitals in western China, which experienced extreme weather in the same year, lends credibility to our study. By contrast, we were able to identify the risk factors for a poor prognosis in patients with heart failure, as well as the variations in clinical characteristics and treatment approaches between the groups with excellent and poor prognoses. The distinctions between CHS and EHS, as well as the differences in survival rates between the two groups, were also examined. Our research will provide some recommendations for the clinical treatment and prevention of HS. Furthermore, our work will fill a knowledge gap regarding HS in western China and serve as a benchmark for future research.

The study has several limitations. Firstly, our multicenter retrospective study, which focused on the western region, may have limited generalizability due to weather and topographic differences in that area. Secondly, variations in local economic conditions and uneven allocation of healthcare resources may lead to differences in treatment approaches, thereby affecting the prognosis of different patients. Lastly, our study lacked mechanistic insights, and future research should delve deeper into the mechanisms and incorporate meteorological features.

## Conclusion

According to our research, individuals with psychosis, hypertension, diabetes, the elderly, and males are more susceptible to developing HS. High blood cell counts, liver and renal damage, and abnormal coagulation may indicate a poor prognosis. In patients with heart failure, APACHE II, Lac, and Scr were independent risk factors for a poor prognosis. Predicting a poor prognosis becomes more accurate when these three indicators are combined. Impaired organ function is a manifestation of heatstroke affecting the body. Furthermore, the mortality rate among CHS patients is higher than that among EHS patients. These findings may provide valuable insights for the therapeutic management and prevention of HS, enabling medical professionals to treat patients more effectively and improve their prognosis.

## Data Availability

The raw data supporting the conclusions of this article will be made available by the authors, without undue reservation.
