# Supplementary material for: Analysis of clinical characteristics and risk factors for patients with heat stroke in western China in 2022: a multicenter retrospective study
Source: Front Med (Lausanne). 2025 Jan 22;12:1467771. doi: 10.3389/fmed.2025.1467771 (PMC11794796; doi:10.3389/fmed.2025.1467771)
Supplement: Supplementary file 1 [file Data_Sheet_1.docx]

ONLINE SUPPLEMENTAL CONTENT

Analysis of clinical characteristics and risk factors for patients with heat stroke in western China, Sichuan and Chongqing in 2022: a multicenter retrospective study

CONTENTS

[Supplemental Table 1. Comparison of the treatment modalities between the different groups of patients 1](#_Toc155618963)

[Supplemental Table 2. Multifactor logistic regression for the poor prognosis group 3](#_Toc155618964)

[Supplemental Table 3. Survival state in the CHS group and EHS group 4](#_Toc155618965)

[Supplemental Figure 1. Study sites of urban residents in Sichuan Province and Chongqing city 5](#_Toc155618966)

[Supplemental Figure 2. Number of patients by age group 6](#_Toc155618967)

[Supplemental Figure 3. Survival from admission to day 28 according to Kaplan–Meier analysis with the log-rank test. 7](#_Toc155618968)

[Chinese ethical review 8](#_Toc155618969)

[English ethical review 9](#_Toc155618970)

Supplemental Table 1. Comparison of the treatment modalities between the different groups of patients

|  | Good prognosis  (n=198) | Poor prognosis  (n=49) | *P* value | CHS  (n=218) | EHS  (n=29) | *P* value |
| --- | --- | --- | --- | --- | --- | --- |
| Antibiotic | 151(76.3%) | 42(85.7%) | 0.152 | 169(77.5%) | 24(82.8%) | 0.522 |
| Hypothermia | 165(83.3%) | 36(73.5%) | 0.112 | 177(81.2%) | 24(82.8%) | 0.839 |
| Fluid replacement(ml) | 1976(775,2817) | 2955(1500,4550) | ＜0.001* | 2000(1000,3184) | 2354(500,3065) | 0.994 |
| Blood purification | 10(5.1%) | 9(18.4%) | 0.002* | 17(7.8%) | 2(6.9%) | 1 |
| Sedate | 86(43.4%) | 39(79.6%) | ＜0.001* | 109(50.0%) | 16(55.2%) | 0.601 |
| Endotracheal intubation | 61(30.8%) | 39(79.6%) | ＜0.001* | 90(41.3%) | 10(34.5%) | 0.483 |
| Anticoagulant | 63(32.0%) | 18(36.7%) | 0.526 | 74(33.9%) | 7(25.0%) | 0.343 |
| Coagulation factor | 17(8.6%) | 22(44.9%) | ＜0.001* | 33(15.1%) | 6(20.7%) | 0.441 |
| Glucocorticosteroid | 49(24.7%) | 19(38.8%) | 0.049 | 58(26.6%) | 10(34.5%) | 0.372 |
| Enteral nutrition | 76(38.4%) | 26(46.9%) | 0.274 | 88(40.4%) | 11(37.9%) | 0.801 |
| Mannitol | 35(18.4%) | 19(42.2%) | 0.001* | 43(20.8%) | 11(39.3%) | 0.029* |

Fluid replacement indicates the amount of fluid resuscitation on the first day. * Indicates a *P* value＜0.05 compared with the good prognosis group or EHS group.

Supplemental Table 2. Multifactor logistic regression for the poor prognosis group

|  | B value | Exp(B) | Wald value | P value | 95% CI |
| --- | --- | --- | --- | --- | --- |
| Sex | -0.478 | .620 | 0.487 | 0.485 | (0.162-2.371) |
| Age | 0.016 | 1.016 | 0.394 | 0.530 | (0.967-1.068) |
| Lac | 0.254 | 1.289 | 5.787 | 0.016* | (1.048-1.584) |
| Scr | 0.005 | 1.005 | 4.242 | 0.039* | (1.000-1.010) |
| AST | 0.001 | 1.001 | 0.657 | 0.418 | (0.999-1.004) |
| ALT | -0.002 | .998 | 0.469 | 0.494 | (0.991-1.004) |
| CKMB | 0.014 | 1.014 | 3.505 | 0.061 | (0.999-1029) |
| PT | 0.090 | 1.094 | 0.716 | 0.397 | (0.888-1.348) |
| APTT | 0.073 | 1.076 | 2.749 | 0.097 | (0.987-1.174) |
| D-D | 0.000 | 1.000 | 0.442 | 0.506 | (1.000-1.000) |
| PLT | -0.002 | .998 | 0.256 | 0.613 | (0.990-1.006) |
| T | 0.260 | 1.296 | 1.339 | 0.247 | (0.835-2.013) |
| APACHE II | 0.200 | 1.221 | 15.753 | ＜0.001* | (1.016-1.347) |

* Indicates a *P* value＜0.05. D-D indicate D-dimer.

Supplemental Table 3. Survival state in the CHS group and EHS group

| Survival state |  | CHS | EHS | *P* value |
| --- | --- | --- | --- | --- |
| YES |  | 181(83%) | 29(100%) | 0.011* |
| NO |  | 37(17%) | 0(0%) |  |

The mortality rate in the CHS group was significantly higher than that in the EHS group. * Indicates a *P* value＜0.05.


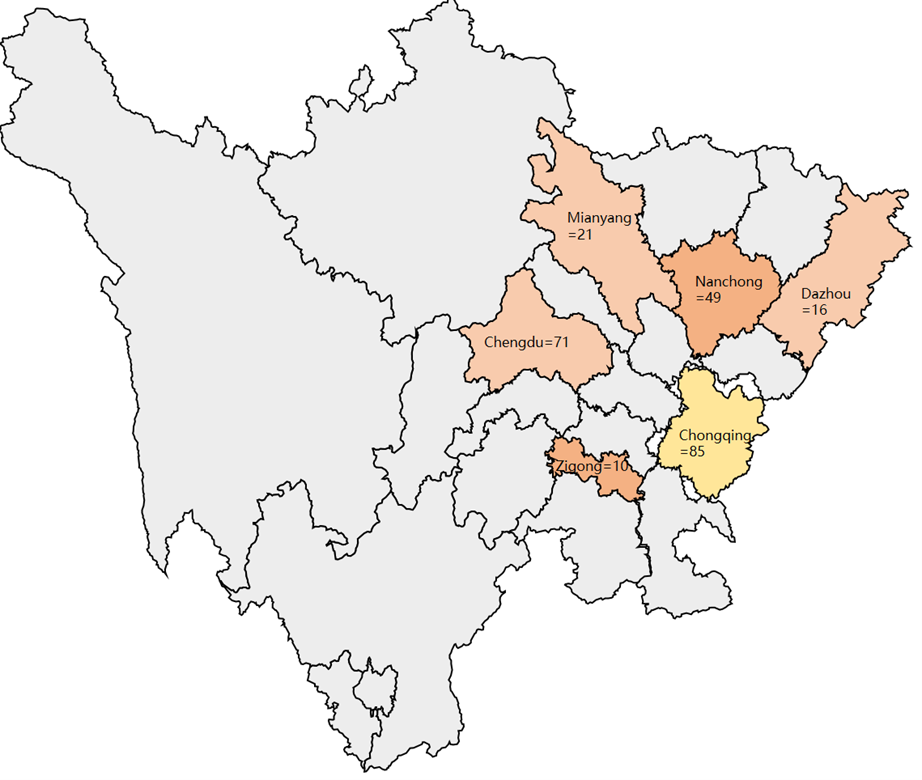


Supplemental Figure 1. Study sites of urban residents in Sichuan Province and Chongqing city


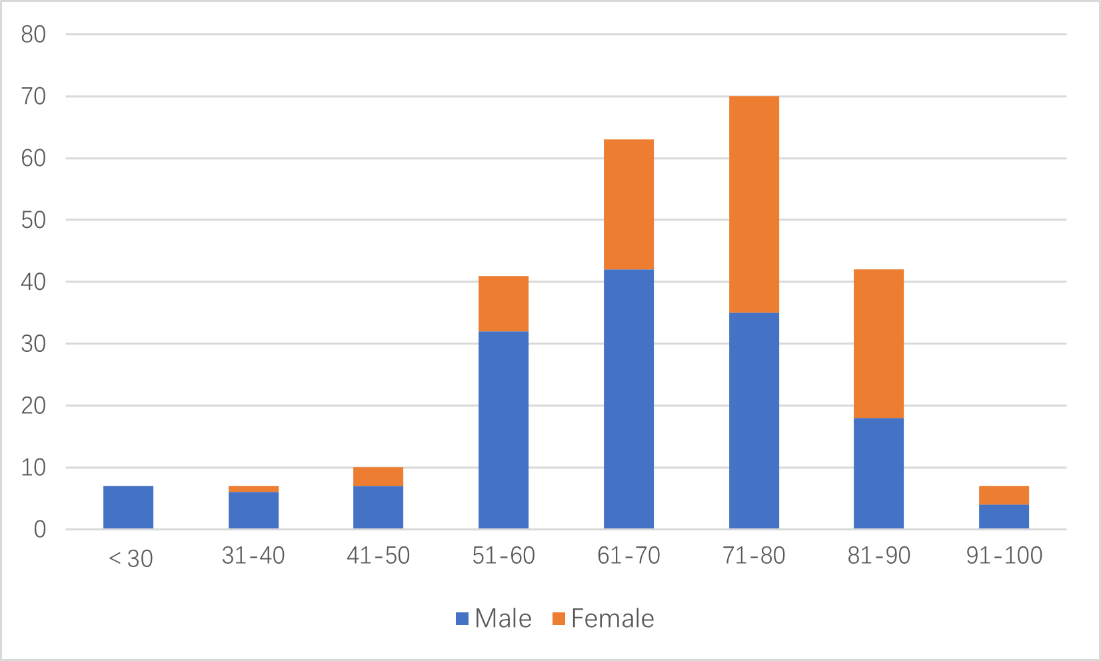


Supplemental Figure 2. Number of patients by age group

The mean age of the patients (standard deviation) was 68.07±14 years, and the percentage of males was 61.5% (152/247). Most of patients were in the 51-90 age group, with the highest number of patients in the 61-80 age group. The number of heatstroke patients younger than 50 years and older than 90 years decreased significantly


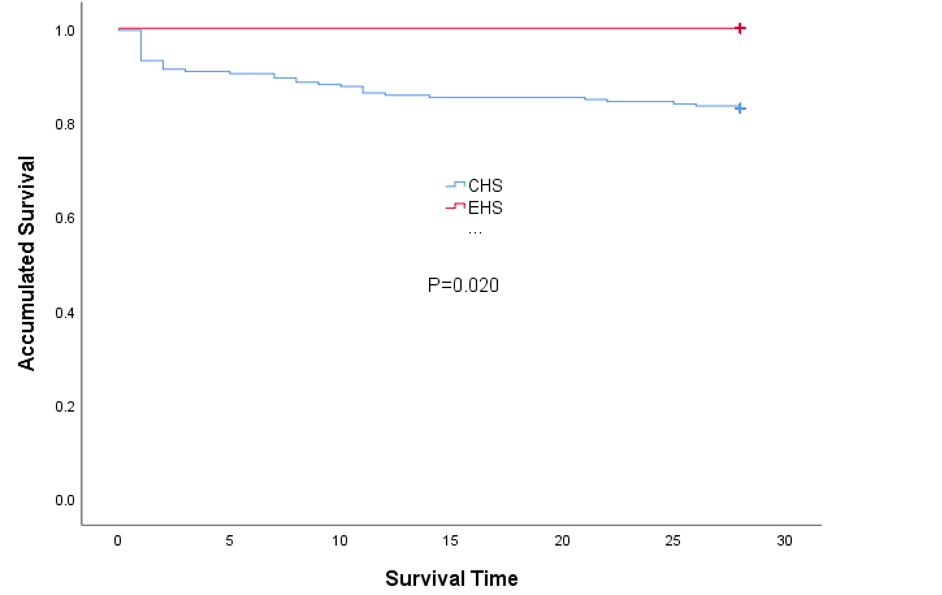


Supplemental Figure 3. Survival from admission to day 28 according to Kaplan–Meier analysis with the log-rank test.

Patients in CHS group had shorter survival time and worse prognosis than those in EHS group

Chinese ethical review


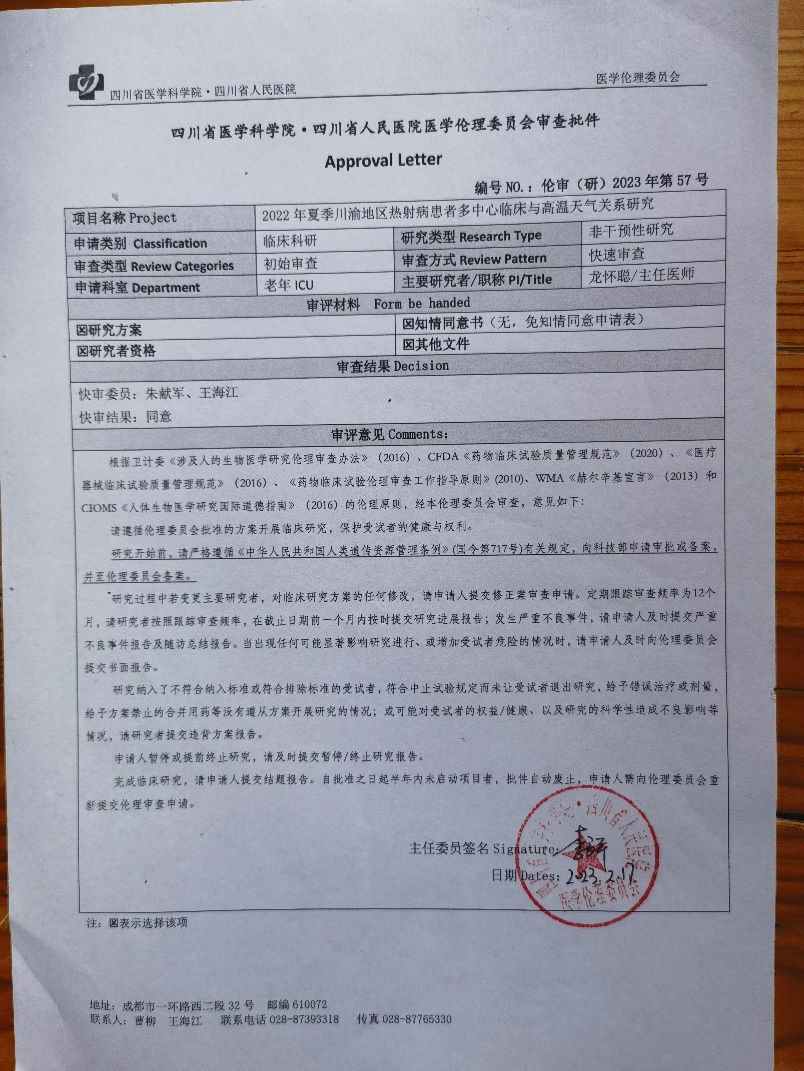


English ethical review

Approval for review by the Medical Ethics Committee of Sichuan Academy of Medical Sciences-Sichuan Provincial People's Hospital

Approval Letter

No. Ethics Review 2023-57

| Project | Study on the relationship between multi-center clinic and high temperature weather in the summer of 2022 in Sichuan-Chongqing area | | |
| --- | --- | --- | --- |
| Classification | Clinical research | Research Type | Non-interventional research |
| Review Categorie |  | Review Pattern | Expedited review |
| Department |  | PI/Title | Huaicong Long/ Archiater |
| Form be handed | | | |
| ☒Research programme | | ☒Informed consent form (No, no informed consent form) | |
| ☒Researcher qualification | | ☒Other files | |
| Decision | | | |
| Expedited review committee: Xianjun Zhu, Haijiang Wang  Result of expedited review: Agreed | | | |
| Comments | | | |
| According to the Measures for Ethical Review of Biomedical Research Involving Human Beings (2016) of the Health Planning Commission, the CFDA Code for Quality Management of Drug Clinical Trials (2020), the Code for Quality Management of Clinical Trials of Medical Devices (2016), the Guiding Principles for Ethical Review of Pharmaceutical Clinical Trials (2010), the WMA Declaration of Helsinki (2013), and the ethical principles of the CIOMS International Ethical Guidelines for Human Ethical Principles for Biomedical Research (2016), reviewed by this Ethics Committee, the opinions are as follows:  Please follow the protocol approved by the Ethics Committee to conduct clinical research and protect the health and rights of the subjects.  Before the commencement of the study, please strictly follow the relevant provisions of the Regulations of the People's Republic of China on the Management of Human Genetic Resources (National Decree No. 717), apply for approval or filing with the Ministry of Science and Technology, and go to the Ethics Committee for filing.  If there is a change of principal investigator during the study and any modification to the clinical study protocol, the applicant is requested to submit an application for amendment review. The frequency of regular follow-up review is 12 months, and the investigators are requested to submit the study progress report on time within one month before the deadline according to the frequency of follow-up review; in case of serious adverse events, the applicant is requested to submit the report of serious adverse events and the summary report of the follow-up visit in a timely manner. When there is any situation that may significantly affect the conduct of the study or increase the risk to the subjects, the applicant is requested to submit a written report to the Ethics Committee in a timely manner.  The investigator is requested to submit a protocol violation report if the study includes subjects who do not meet the inclusion criteria or meet the exclusion criteria, fails to withdraw subjects from the study if they meet the requirements for discontinuation of the trial, administers the wrong treatment or dosage, administers a combination of medications that are prohibited by the protocol, etc.; or if there may be an adverse effect on the rights/health of the subjects or on the scientific validity of the study.  The applicant suspends or terminates the study early, please submit the suspension/termination report in a timely manner.  Upon completion of the clinical study, the applicant is requested to submit a completion report. If the project is not initiated within six months from the date of approval, the approval will be automatically revoked and the applicant is required to resubmit the application for ethical review to the Ethics Committee.  Signature: Liangping LI  Dates: 17/2/2023 | | | |

Tips:☒Indicates select item

Address: No.32, West 2nd Section, 1st Ring Road, Chengdu, China. Postal code: 610072

Contactor: Liu Cao, Haijiang Wang. Tel: 028-87393318 Fax: 028-87765330
